# AMH Is a Good Predictor of Metabolic Risk in Women with PCOS: A Cross-Sectional Study

**DOI:** 10.1155/2021/9511772

**Published:** 2021-08-12

**Authors:** Miaoxian Ou, Pei Xu, Han Lin, Kaichi Ma, Mingxing Liu

**Affiliations:** ^1^Department of Obstetrics and Gynecology, Key Laboratory for Major Obstetric Diseases of Guangdong Province, The Third Affiliated Hospital of Guangzhou Medical University, Guangzhou, China; ^2^Department of Gynecology of Traditional Chinese Medicine, The First Affiliated Hospital of Guangzhou Medical University, Guangzhou, China

## Abstract

**Objective:**

The relationship between metabolic risk and ovarian function is ambiguous. This retrospective study analyzed the medical records of 461 PCOS patients collected between January 2019 and June 2020 to investigate the relationship between serum anti-Müllerian hormone (AMH) and parameters of metabolic risk in the population with polycystic ovary syndrome (PCOS).

**Methods:**

A total of 461 PCOS patients aged 20–40 years were included and stratified into four groups according to the AMH level. The association between AMH and the parameters related to metabolic risk in these groups was compared, and the discrepancies were further explored. Binary logistic regression was performed to examine the risk factors of HOMA-IR. The values of AMH that best predicted the risk of HOMA-IR were also analyzed by ROC curves.

**Results:**

AMH was negatively associated with HOMA-IR (odds ratio (OR) −0.279, 95% confidence interval (CI) −0.36 to −0.20), fasting insulin (OR −0.282, 95% CI −0.36 to −0.20), 1-hour postprandial insulin (OR −0.184, 95% CI −0.28 to −0.11), 2-hour postprandial insulin (−0.180, 95%CI −0.28 to −0.11), 3-hour postprandial insulin (OR −0.198, 95% CI –0.30 to −0.13), waist-hip ratio (OR −0.235, 95% CI −0.31 to −0.14), and body mass index (OR −0.350, 95% CI −0.43 to −0.27). There was no statistically significant relationship between blood pressure, serum glucose profile, or lipid levels and AMH. Binary logistic regression showed that AMH protected against the occurrence of PCOS patients (OR: 0.835, 0.776, and 0.898). For the prediction of HOMA-IR, AMH had an AUC-ROC of 0.704 (95% CI 0.652–0.755) with a cutoff value of 7.81 mmol/L, a sensitivity of 70.3%, and a specificity of 70.1%.

**Conclusions:**

Higher AMH levels were significantly associated with a lower insulin profile and might be a useful predictor for HOMA-IR in PCOS patients.

## 1. Introduction

Polycystic ovary syndrome (PCOS) is a heterogeneous endocrinopathy affecting 5–12% of women of reproductive age [1]. The basic characteristics include ovulatory dysfunction, hyperandrogenism, and polycystic ovary morphology (PCOM). About half of all PCOS patients have metabolic and hormonal derangements, which increases their risk of cardiometabolic disease and reduces their overall quality of life [2]. Obesity, dyslipidemia, hypertension, hyperglycemia, and insulin resistance are all associated with PCOS and contribute to the mortality and morbidity in PCOS patients [3]. As the ovaries age, their functions might decrease and this can lead to increased risk for metabolic disorders, which is similar to what is seen in normal reproductive aging and the onset of menopause [4]. The association between decreased ovarian reserve and increased risk for metabolic disease might be strong among women over time, but the relationship between ovarian reserve and metabolism among PCOS populations is controversial and remains unsolved in current studies [5, 6]. Therefore, it is essential to identify specific ovary function biomarkers to predict the risk of metabolic disorders among PCOS patients.

Anti-Müllerian hormone (AMH) is a glycoprotein hormone secreted by the granulosa cells of small antral follicles within the ovary [7]. As a stable reflector of the primordial follicle pool, AMH has recently emerged as the most promising biomarker of ovarian reserve quantification, and higher serum AMH levels have been shown to be associated with hyperactivity of primordial follicles and greater ovarian stimulation [8]. Women with PCOS have higher AMH levels compared to healthy women, and AMH levels decline as the women age [9], and it has been reported that higher AMH levels are correlated with reproductive disorders such as menstrual dysfunction and resistance to ovulation induction [10, 11]. The functions of AMH beyond reproduction have been investigated recently, but no strong conclusions could be drawn regarding the relationship between AMH and metabolic health in PCOS patients due to the heterogeneous nature of such populations and the lack of adjustment for possible confounders of metabolism. In healthy adult women with a regular menstrual cycle, lower AMH levels have been found to be associated with increased risk of metabolic disorders, although this correlation is negative in adolescents [12]. There is also some evidence indicating that lower AMH level is a metabolic risk factor, but debates on the correlation between AMH and obesity emphasize the complexity of the physiopathology of the PCOS population [6, 13].

Thus, the question remains as to the relationship between AMH levels and metabolic function in women with PCOS. We hypothesized that AMH predicts the risk of metabolic disorders after adjusting for age and body mass index (BMI) in PCOS patients.

## 2. Materials and Methods

### 2.1. Subjects

In this retrospective cohort study, we enrolled patients diagnosed with PCOS who were referred to the infertility clinic of the Third Affiliated Hospital of Guangzhou Medical University, Guangzhou, China, between January 2019 and June 2020. The participants' information was extracted from an electronic medical database, and written informed consent was obtained from these participants. The study was conducted in accordance with the World Medical Association Declaration of Helsinki. Medical charts of all patients were reviewed, and 461 patients were included according to the inclusion and exclusion criteria.

Women who were diagnosed with PCOS by the revised Rotterdam 2003 criteria were included. The diagnosis should include at least two of the three criteria of oligomenorrhea and/or amenorrhea, clinical hyperandrogenism and/or hyperandrogenemia, and PCOM and exclude other etiologies [14]. Oligomenorrhea refers to a menstrual interval of 35–182 days, while amenorrhea refers to the absence of vaginal bleeding for more than 182 days [14]. Clinical hyperandrogenism was considered present if the Ferriman–Gallwey score was ≥4, and hyperandrogenemia was defined as a free androgen index (FAI) >6.5 [15]. PCOM was defined as 12 or more follicles measuring 2–9 mm in diameter in either ovary and/or an ovarian volume of more than 10 ml by transvaginal or transrectal ultrasonography [14]. The exclusion criteria were as follows: (1) age <20 years old or >40 years old, (2) other diseases causing oligomenorrhea, amenorrhea, or hyperandrogenic disorders including thyroid disorders, hyperprolactinemia, congenital adrenal hyperplasia, androgen secreting tumors, or Cushing syndrome, (3) a history of gynecological surgery or pelvic radiation therapy, and (4) taking oral contraceptives, steroid hormone drugs, hypoglycemic agents, antilipemic agents, antihypertensive drugs, or other drugs influencing metabolism, such as metformin, statins, and so on, in the past six months. The included women were stratified into four groups according to the <25th, 25th to 50th, 50th to 75th, and >75th percentile of serum AMH concentration.

### 2.2. Anthropometric Measurements

Body height and weight were measured by a digital scale and a stadiometer to the nearest 0.1 cm and 0.1 kg, respectively. The waist circumference (WC) and hip circumference (HC) were measured to the nearest 0.1 cm by placing a measuring tape in a horizontal position between the lower rib margin and the iliac crest. Blood pressure (BP), including systolic blood pressure and diastolic blood pressure, was measured twice in a seated and quiet position with a standard mercury sphygmomanometer, and the mean result was recorded.

### 2.3. Biochemical Assays

Blood samples were taken between 9:00 a.m. and 11:00 a.m. after a 12-hour overnight fast during days 2–5 of the spontaneous menstrual cycle. Patients with amenorrhea were sampled at any time. All patients then received 75 g oral glucose and blood samples were collected at 0, 60, 120, and 180 min. Serum AMH follicle-stimulating hormone (FSH), luteinizing hormone (LH), estradiol (E2), progesterone (Prg), total testosterone (TT), prolactin (PRL), and sex hormone-binding globulin (SHBG) were measured by the chemiluminescence method with a between-batch coefficient of variation of 15%. Hormone assays have been described in detail elsewhere [16]. High-density lipoprotein cholesterol (HDL-C), low-density lipoprotein cholesterol (LDL-C), total cholesterol (TC), triglyceride (TG), fasting plasma glucose (FPG), fasting insulin (FINS), postprandial glucose (PPG), and postprandial insulin (PPI) were all assessed by enzymatic colorimetric assay. All measurements were made with an Elecsys Autoanalyzer (Roche). All blood samples were stored at 4°C on the day of collection and were taken simultaneously at the same laboratory.

### 2.4. Definitions

BMI was calculated as body weight/(height)^2^. Waist-hip ratio (WHR) was calculated as WC/HC. The index of homeostasis model assessment of insulin resistance (HOMA-IR) was calculated as HOMA-IR = (FPG × FINS)/22.5, and FAI was calculated as FAI = TT/SHBG × 100. Insulin resistance was defined as HOMA-IR ≥2.5 [17].

### 2.5. Statistical Analysis

All statistical analyses were performed by SPSS version 24.0 (SPSS Inc., Chicago, IL, USA). Clinical data in the form of continuous variables were presented as the mean ± SD for normally distributed data or as the median and interquartile range for skewed data and were compared by analysis of covariance. Categorical variables are presented as percentages and were compared using chi-square tests. Pearson's correlation analysis was used to analyze the correlations between AMH and all metabolic parameters, and binary logistic regression was used to examine independent predictors of HOMA-IR. The results are expressed as adjusted odds ratio (OR) with 95% confidence intervals (CIs). The receiver operating characteristic (ROC) curve was used to identify the best cutoff value of AMH for predicting HOMA-IR in PCOS patients. All statistical tests were two-sided, and a *p* value of <0.05 was considered statistically significant.

## 3. Results

Data from 681 patients were recorded, and 461 patients were selected for statistical analysis based on the inclusion and exclusion criteria. There were 31.24% (144/461) of patients who were diagnosed with insulin resistance. The general anthropometric characteristics and metabolic characteristics are presented in Table 1 separated into four subgroups according to the quartile of serum AMH. The mean age was 27.44 years old (20–38 years old), with a median BMI of 22.48 kg/m^2^ (13.34–41.40 kg/m^2^). The overall median AMH was 9.53 ng/ml (1.76–18 ng/ml). There were 144 patients (31.24%) who were diagnosed with insulin resistance. BMI and WHR decreased and the mean antral follicle count (AFC) rose as AMH increased (*p* < 0.05). No statistically significant differences were found between levels of AMH regarding age, BP, HR, WC, HC, or lifestyle including smoking, drinking, and exercise (*p* > 0.05). Higher AMH levels tended to be correlated with lower age, but these differences were not statistically significant.

The biochemical parameters including serum LH level, FINS, 1 h-PPI, 2 h-PPI, 3 h-PPI, and HOMA-IR decreased as AMH rose, and these differences were statistically significant (*p* < 0.05) (Table 1). No significant association was found between Prg, FSH, PRL, SHBG, FAI, TSH, lipids, or blood glucose and AMH (*p* > 0.05). AMH increased as TT increased, but this was not statistically significant (*p* > 0.05).

**Figure 1:**
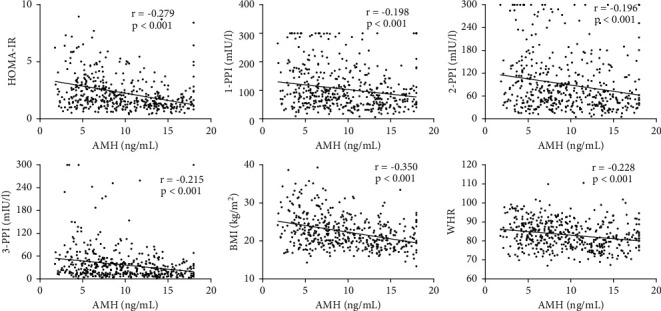
Relationship between AMH and metabolic parameters in women with PCOS.

The relationships between AMH and metabolic parameters that were found to be significant in Table 1 are shown in Figure 1. According to Pearson's correlation analysis, AMH levels were significantly negatively correlated with BMI (*r* = –0.350, *p* < 0.001), WHR (*r* = –0.235, *p* < 0.001), HOMA-IR (*r* = –0.279, *p* < 0.001), FINS (*r* = –0.282, *p* < 0.001), 1 h-PPI (*r* = –0.184, *p* < 0.001), 2 h-PPI (*r* = –0.180, *p*=0.001), and 3 h-PPI (*r* = –0.198, *p* < 0.001).

In addition, binary logistic regression was also performed to examine the predictive factors of HOMA-IR (Table 2). For women with PCOS, AMH was a protective factor for HOMA-IR (OR 0.873, CI% 0.794–0.883, *p* < 0.001), and this was still significant after adjusting for age and BMI (OR 0.895, 95 CI% 0.842–0.895, *p* < 0.001). Further multiple logistic regression adjusting for age, BMI, lifestyle (exercise, smoking, and drinking), WHR, FAI, LH, and AFC showed that AMH remained an independent protective factor against elevated HOMA-IR in PCOS patients (OR 0.835, 95 CI% 0.776–0.898, *p* < 0.001).

**Figure 2:**
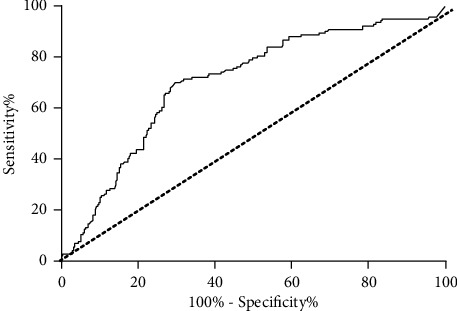
Receiver operating characteristic (ROC) curves for the prediction of HOMA-IR by serum AMH (ng/mL).

The accuracy of AMH for predicting HOMA-IR in PCOS subjects was evaluated as shown in Figure 2. The identified cutoff values for AMH were 7.81 mmol/L with a sensitivity of 70.3%, a specificity of 70.1%, and an area under the curve of 0.704 (95% CI 0.652–0.755, *p* < 0.001).

## 4. Discussion

In the current study, we found that there was a negative relationship between AMH and BMI, WHR, and insulin level but no correlation with BP, lipid, or glucose levels among PCOS patients. In addition, AMH acted as a protective factor against the occurrence of insulin resistance in PCOS patients and PCOS patients with a AMH level lower than 7.81 mmol/L might be a predictor of increased risk for insulin resistance. The overall differences in these parameters were consistent with our hypothesis that lower AMH level is significantly associated with severe insulin resistance and might be useful for predicting elevated HOMA-IR in PCOS patients.

BMI was strongly associated with AMH level in our study, and women with higher BMI were shown to have lower AMH levels. However, the mean BMI was in the normal and overweight range and not in the obesity range in our study of ethnic and regional differences. In contrast to our study, some studies have shown no link between AMH and BMI, but these studies only had small sample sizes and did not show statistically significant differences [13, 18]. Similar to our study, BMI was believed to act as a confounder between risk of metabolic disorders and ovarian reserve among PCOS patients in most studies [19, 20]. Women with higher BMI might be more susceptible to developing metabolic disorders than those with lower BMI among both PCOS and non-PCOS populations [6, 20]. The effect of BMI on AMH might be explained by a toxic effect of adiposity on granulosa cell function because of AMH production or due to a diluting effect on serum AMH levels. This notion is supported by an experiment showing that mice exposed to high levels of lipids had increased levels of apoptosis in the ovary, and it is plausible that BMI plays an important role in this [21].

Higher AMH levels were associated with decreased risk of insulin resistance in our study, and AMH can further be the predictive factor of insulin resistance. In some studies, no association between AMH and metabolism disorders, including insulin resistance, was found among adolescents because few cardiometabolic disorders occur in such populations, while inconsistent results were seen in adults [22]. In some small-sample studies, AMH seemed not to be correlated with HOMA-IR in adult females with irregular periods, but other factors were not elucidated further [6]. The ethnic populations, clinical setting, and inclusion criteria may contribute to the discrepancies among these studies. Confounding factors, which can contribute to the metabolic risks, including age, smoking, drinking, and exercise, were not statistically different in our study, but none of these lifestyle factors were adjusted for in previous studies. The association between insulin resistance, one of the most important factors in PCOS patients, and AMH might be explained by hyperinsulinemia altering intraovarian AMH signaling and depressing the activity of granulosa cells [23]. Another possible pathophysiological theory might be end-organ hypoxia because hyperinsulinemia damages the vascularization of the ovaries, reduces ovarian blood flow, and leads to accelerated ovarian decline, which can be reflected by the concentration of AMH [24].

The glucose profile did not share the same correlation with AMH as insulin in our study, which might suggest that glucose compounds work through a different pathway than insulin in PCOS patients. Other biomarkers of metabolism such as lipids and BP were also not correlated with AMH. One of the reasons for this might be that the population of our study was in the normal and overweight range and not obesity range and would thus be expected to have relatively normal lipid levels. Another reason might be that this was a cross-sectional study and only a short period of time was probed. A longitudinal study conducted on 1015 PCOS patients showed that lipid profiles might not differ at initiation when they were young, but lipid deterioration occurs in women with low ovarian reserve as time progresses [25].

There was also a positive correlation between AMH and other hormone profiles such as LH and TT, which was in line with the previous study that LH and TT rose as AMH increased [26]. Thus, we adjusted these confounders in our logistic regression model, and the result remained that AMH was a protective factor against elevated HOMA-IR. In mouse models, AMH was found to act as a central regulator of the hypothalamic-pituitary-adrenal axis (HPA axis), which could increase gonadotropin-releasing hormone neuronal activation and neurohormone secretion [8]. Elevated AMH can lead to an increase in LH through gonadotropin-releasing hormone activation, which in turn promotes the production and secretion of ovarian androgens by theca cells in the presence of insulin [27]. Hyperandrogenism can also modulate insulin sensitivity, which may be the key to why insulin resistance was found to be associated with AMH [28, 29]. Jie et al. found that PCOS with hyperandrogenism is associated with a high rate of nonalcoholic fatty liver disease, which can reduce the secretion of SHBG and in turn lead to a higher FAI [30].

The major strength of this study is the explicit risk assessment between AMH and common metabolism indexes in PCOS patients. The complex relationship between metabolic risk and ovarian function limits the exploration of the mechanism and treatment of PCOS patients, and a better understanding of these mechanisms will contribute to more accurate evaluations of the metabolic risks associated with PCOS. Early prediction of metabolic disorders can lead to earlier interventions to ameliorate further comorbidities and to protect ovarian function in PCOS patients. One limitation of this study is that it was a cross-sectional, retrospective, single-center study, which may have a degree of bias and lack of representativeness, and such a study cannot provide any evidence for causality. However, the basic characteristics and factors that influence metabolism were not statistically different, so the bias may have been minimized. In addition, a multivariate analysis was carried out to determine further relationships between the factors. Further interventional studies and experimental explorations are needed to have an insight into the interplay between AMH and metabolic changes in order to elucidate the pathophysiology behind the findings presented here.

## 5. Conclusion

In conclusion, this study characterized a population of PCOS patients with different AMH levels. Increasing AMH was a protective factor against elevated insulin resistance, and a serum AMH concentration of less than 7.81 mmol/L might be a useful predictor of increased risk for insulin resistance. In addition, BP and serum glucose and lipid levels were not found to be related to AMH. Early prediction of metabolic disorders can help to intervene against further comorbidities, and protecting ovarian function would help to prevent metabolic diseases in women with PCOS.

## Figures and Tables

**Table 1:** General demographic and metabolic characteristics of the participants in the four quartiles of AMH.

	1st quartile (*n* = 115)	2nd quartile (*n* = 116)	3rd quartile (*n* = 116)	4th quartile (*n* = 114)	*p*
AMH	4.45 ± 0.97	7.43 ± 0.79	10.55 ± 1.09	15.26 ± 1.74	<0.05
Mean AFC	12.97 ± 2.69	14.55 ± 2.93	15.68 ± 3.07	17.30 ± 4.49	<0.05
*Anthropometric parameters*					
Age	28.09 ± 3.96	27.53 ± 4.09	27.12 ± 3.96	27.00 ± 4.11	0.166
BMI	24.27 ± 5.30	23.28 ± 4.24	21.60 ± 3.36	20.54 ± 3.45	<0.05
SBP	108.56 ± 12.58	109.85 ± 13.19	106.64 ± 11.84	106.60 ± 11.25	0.12
DBP	73.75 ± 10.50	73.93 ± 9.89	71.96 ± 8.73	71.99 ± 20	0.21
WHR	85.64 ± 7.04	84.11 ± 7.31	81.72 ± 6.77	81.47 ± 6.98	<0.05

*Lifestyle parameters*					
Smoker	45.5% (5)	18.2% (2)	27.3% (3)	9.1% (1)	0.354
Drinker	25.0% (13)	21.2% (11)	32.7% (17)	21.2% (11)	0.573
Exercise					0.064
Never	21.2% (46)	24.0% (52)	24.4% (53)	30.4% (66)	
1–3 times/month	29.8% (25)	26.2% (22)	19.0% (16)	25.0% (21)	
>1-2 times/week	27.4% (44)	26.3% (42)	29.4% (47)	16.9% (27)	

*Hormone parameters*					
FSH	5.71 ± 1.62	5.58 ± 1.46	5.75 ± 1.46	6.05 ± 1.67	0.144
LH	7.60 ± 4.79	9.00 ± 4.79	11.16 ± 5.56	12.91 ± 6.83	<0.05
E_2_	150.06 ± 55.82	159.87 ± 51.77	161.39 ± 61.53	169.52 ± 58.15	0.84
Prg	0.73 ± 0.49	0.81 ± 0.62	0.93 ± 0.62	0.77 ± 0.69	0.418
PRL	16.74 ± 10.13	17.22 ± 11.88	18.86 ± 11.79	16.26 ± 8.67	0.280
TT	1.17 ± 0.70	1.27 ± 0.66	1.66 ± 0.62	1.74 ± 0.78	<0.05
SHBG	62.65 ± 48.20	66.61 ± 52.45	70.07 ± 53.84	74.57 ± 60.94	0.38
FAI	3.56 ± 4.53	3.52 ± 3.24	3.61 ± 2.87	4.03 ± 4.19	0.725
TSH	1.85 ± 0.94	1.80 ± 0.95	2.01 ± 1.38	1.69 ± 0.84	0.139

*Metabolic parameters*					
HOMA-IR	3.18 ± 3.09	2.41 ± 1.36	1.87 ± 1.14	1.75 ± 1.32	<0.05
Yes	41.0% (59)	32.6% (47)	16.0% (23)	10.4% (15)	<0.05
No	17.7% (56)	21.8% (69)	29.3% (93)	31.2% (99)	
FINS	13.78 ± 11.93	10.81 ± 5.75	8.61 ± 4.81	8.02 ± 5.37	<0.05
1 h-PPI	125.68 ± 80.85	106.52 ± 66.22	96.64 ± 64.44	85.46 ± 61.64	<0.05
2 h-PPI	113.83 ± 84.93	89.84 ± 71.00	85.91 ± 66.37	71.96 ± 62.82	<0.05
3 h-PPI	49.26 ± 56.33	42.72 ± 46.52	33.13 ± 32.99	25.05 ± 31.47	<0.05
FPG	5.13 ± 1.64	4.95 ± 0.58	4.84 ± 0.73	4.81 ± 0.56	0.051
1 h-PPG	8.49 ± 2.24	8.36 ± 2.25	7.91 ± 2.21	7.99 ± 2.11	0.137
2 h-PPG	7.14 ± 2.23	6.69 ± 1.91	6.73 ± 4.62	6.28 ± 1.68	0.164
3 h-PPG	4.83 ± 1.52	4.70 ± 1.26	4.72 ± 1.13	4.74 ± 1.21	0.870
HDL-C	1.93 ± 0.97	1.91 ± 0.88	2.15 ± 0.88	2.03 ± 0.74	0.138
LDL-C	2.34 ± 1.06	2.46 ± 0.98	2.50 ± 1.03	2.48 ± 0.90	0.637
TC	4.58 ± 1.25	4.55 ± 0.80	4.58 ± 0.75	4.58 ± 0.76	0.787
TG	1.27 ± 0.74	1.30 ± 1.25	1.05 ± 0.57	1.22 ± 2.37	0.548

AMH, anti-Müllerian hormone; AFC, antral follicle count; BMI, body mass index; SBP, systolic blood pressure; DBP, diastolic blood pressure; WHR, waist-hip ratio; FSH, follicle-stimulating hormone; LH, luteinizing hormone; E_2_, estradiol; Prg, progesterone; PRL, prolactin; TT, total testosterone; SHBG, sex hormone-binding globulin; FAI, free androgen index; FINS, fasting insulin; PPI, postprandial insulin; FPG, fasting plasma glucose; PPG, postprandial glucose; HDL-C, high-density lipoprotein cholesterol; LDL-C, low-density lipoprotein cholesterol; TC, total cholesterol; TG, triglyceride.

**Table 2:** Binary logistic regression with HOMA-IR as the output variable in PCOS patients.

	B	OR	95% CI		*p*
AMH	−0.178	0.837	0.794	0.883	<0.001
Adjusted for age and BMI	−0.115	0.892	0.840	0.947	<0.001
Adjusted for age, BMI, and lifestyle (exercise, smoking, and drinking)	−0.111	0.895	0.842	0.895	<0.001
Multivariate adjusted^*∗*^	−0.175	0.840	0.780	0.904	<0.001

^*∗*^Multiple logistic regression adjusted for age, BMI, lifestyle (exercise, smoking, and drinking), WHR, FAI, LH, and AFC. BMI, body mass index; WHR, waist-hip ratio; FAI, free androgen index; LH, luteinizing hormone; AFC, antral follicle count.

## Data Availability

The data used to support the findings of this study are available from the corresponding author upon request.
